# Notes and News

**Published:** 1896-08-15

**Authors:** 


### Notes and News.

(Collected and Communicated.)

The Queen has appointed Mr. Rickman John Godlee to be Surgeon-in-Ordinary to her Majesty's household in place of Sir Spencer Wells resigned.

Princess Louise opened a bazaar in the park of Crewe Hall last week in behalf of the Crewe Hospital Memorial Fund. As there was a large attendance, it is to be hoped that a good sum was collected.

The Alps are claiming their annual victims as usual this year. Amongst three recent deaths that are recorded is that of Professor Schmal of Berlin, who fell to a great depth when climbing. He had only recently been appointed body physician to the new Shah of Teheran.

It is reported that in the recent Italian war in Africa over 200 native soldiers captured by the Abyssinians were deprived of a foot and a hand each before being set free, and that the Italian Government has sent out an artificial limb-maker to supply the missing limbs.

The nursing at the Aden Hospital has not been regarded as satisfactory for some time. A scheme has now been started for providing it with a sufficient staff of trained nurses, and also for putting the hospital generally on a proper footing. Contributions are asked towards the £9,000 required for the purpose.

The new hospital in connection with the Aberdeen Royal Asylum was opened last week. The buildings have been erected with all the latest ideas of modern requirements. Everything has been done to render the habitation as bright and attractive as possible. It is connected with the main buildings of the asylum by a glazed corridor, which will also act as a winter garden. The lighting is by electricity, and the furniture is comfortable and home-like.

The list of Royal patrons of the fête and fancy fair to be held in June next in aid of the funds of Victoria Hospital for Children has been increased by the names of the Duke of Cambridge and the Duchess of Teck.

It is proposed to hold an industrial exhibition next year at Newcastle on behalf of the Newcastle Infirmary. A guarantee sum of £10,000 is necessary, and it appears likely this will be forthcoming.

Harrod's Stores have handed over £2,000 to Guy's Hospital as the result of the collection and fête held on behalf of that institution by the employés. The money will be devoted to the endowment of two beds.

The Lord Lieutenant of Cambridgeshire, Mr. Alexander Peckover, has given £1,000 to Addenbrooke's Hospital, Cambridge, to defray the cost of a new operating theatre. Mr. Peckover has twice given similar sums to the hospital since he has been Lord Lieutenant of the county.

Last week the Duke of Cambridge visited the Surrey convalescent home at Seaford. He was conducted over the home by Miss Napper, the lady superintendent, and expressed himself much pleased with the arrangement. The home, which was established some seven years ago, possesses an excellent site, and has an uninterrupted view of the sea front.

There is something evidently of the nature of a misunderstanding in connection with the proposed new cottage hospital for Totnes. The result of the matter is, that a generous donor, Mrs. Champernowne, has withdrawn her intention of commemorating her husband's name in connection with the new institution, though she has generously presented the committee with £600 towards the new building, and £200 as an equivalent for a projected site.

The Cannock Chase Collieries have just been provided with a new cottage hospital through the instrumentality of the Vicar of Hednesford and the committee of the Nursing Association of that place. The workpeople employed at the collieries have agreed to contribute weekly sums, which it is hoped will provide for the necessary expenditure. The Hammerwich Cottage Hospital, which is in the same neighbourhood, has proved so useful an institution that there is little doubt the new hospital will be also much appreciated.

An interesting meeting was held at the Meath Home of Comfort last week. This home was established by Lady Meath to provide a home for epileptic women and girls. The institution has proved most useful and successful, and the proceedings of last week were principally interesting through the dedication of the new wing, which will allow for the reception of twenty-eight more patients. In England there is hardly any provision made for this unfortunate class, and the tax upon the Meath Home has been very severe. Not only is the institution delightfully situated, but it is under the charge of a band of enthusiastic workers, whose strenuous labours on their behalf render the home truly one of comfort for the patients.

